# Generative Phenomenology of form perception: Perceptograms and cortical models for amblyopic phantom percepts

**DOI:** 10.1101/2025.06.24.661078

**Published:** 2025-06-25

**Authors:** Akihito Maruya, Bhavatharini Ramakrishnan, Farzaneh Olianezhad, Jingyun Wang, Jose-Manuel Alonso, Qasim Zaidi

**Affiliations:** 1 Graduate Center for Vision Research, State University of New York, New York, NY.

**Keywords:** visual perception, form perception, perceptual distortions, receptive fields, visual disorders, amblyopia

## Abstract

We introduce Generative Phenomenology: making viewable images of people’s perceptions (Perceptograms) and generating the images from neural models, as a powerful technique for understanding the neural bases of perception. Amblyopia, a disorder of spatial vision, provides a perfect case because signals from the two eyes go through partly different cortical neurons, and many amblyopes report phantom forms when viewing sinusoidal gratings through their amblyopic eye (AE) but not through the fellow eye (FE). Using a dichoptic display, we acquired high-fidelity perceptograms for 24 gratings shown to AE while sums-of-gratings plaids were shown to FE with contrast, frequency, phase, and orientation of the plaid gratings adjusted to match the two percepts exactly. Plaids provided exact matches to 92.6% of distortions. A formal equation that the signals generated in visual cortex by the test gratings seen through AE match the signals generated by their matched perceptograms seen through FE for each observer, was used to analytically derive cortical filters processing AE signals as linear transforms of standard steerable filters modeling normal V1 neurons for FE. Passing gratings through AE filters accurately generated the measured perceptograms. The filter transformations reflected complex changes in V1 receptive fields and possibly in cross-correlations. The AE filters also explained amblyopic deficits in perceiving sinusoidally modulated circular contours and were consistent with orientation perceptive fields estimated from reverse-correlation experiments. Changes in neuronal receptive fields thus have profound effects on perception, to the extent that observers can see more features than are present in the viewed stimulus.

## INTRODUCTION

If we could picture the percepts of people (or animals) and generate these images from cortical models (Generative Phenomenology), we could learn so much more than from experimental methods which only provide numerical estimates of parameters. Amblyopia, a disorder of spatial vision, provides an interesting case for a generative study of phenomenology because signals from the two eyes go through partially different cortical neuronal populations. Abnormal neural development in the cortex is a result of imbalance in the input from the two eyes caused by early misalignment of eyes (strabismus), unequal refractive power (anisometropia), or form deprivation (cataract)^1^. The disorder persists despite the restoration of good retinal image quality and eye health and affects 2–3% of the population. Consequently, many amblyopes see distorted versions of forms through the amblyopic eye (AE) but not through the fellow eye (FE). AE distortions can potentially be matched perfectly through the FE by rapidly generating images from a large set and using efficient search procedures. The matched images perform the function of a “Perceptogram”, which is an externally viewable record of an internal perceptual image^2^ or of perceptual distortions of a target image^3^. After obtaining the perceptogram, synthesizing it from generative cortical models can provide detailed insights into cortical processing and neural deficits, and teach us about how forms are created by the brain from the sensory input.

Striking form distortions seen by amblyopes had been noted for letters^4^ and circular shapes^5^, but the most diagnostic distortions were documented by showing high contrast sinusoidal gratings to the AE and drawing the percept through the FE^6,7^. The drawings fell into a few classes: wavy appearance of straight gratings, gratings with abrupt phase shifts orthogonal to the grating orientation, correct grating orientation with an additional lower contrast grating at some oblique orientation, fragmented gratings, and large gaps in the grating. The drawings representing the phantom forms evoked by gratings were shown to resemble plaids formed by sums of pairs of gratings^7^, a key insight reproduced in Supplemental Figure S1. Forms are made of local orientations, which are initially extracted by neurons in primary visual cortex (V1) and then combined in later areas. The deficient processing likely starts with orientation encoding in V1^8^ but also involves later mechanisms that decode orientation defined forms^9^. The resemblance of the drawings to plaids ruled out neural scrambling^10,11^ or systematic shifts in the neural map^12–14^ as explanations. Phantom forms are potentially a bigger visual problem than the loss of 2 lines of visual resolution (the standard definition of amblyopia), so the complete lack of progress in this area in the last 20 years is surprising.

**Figure 1.**
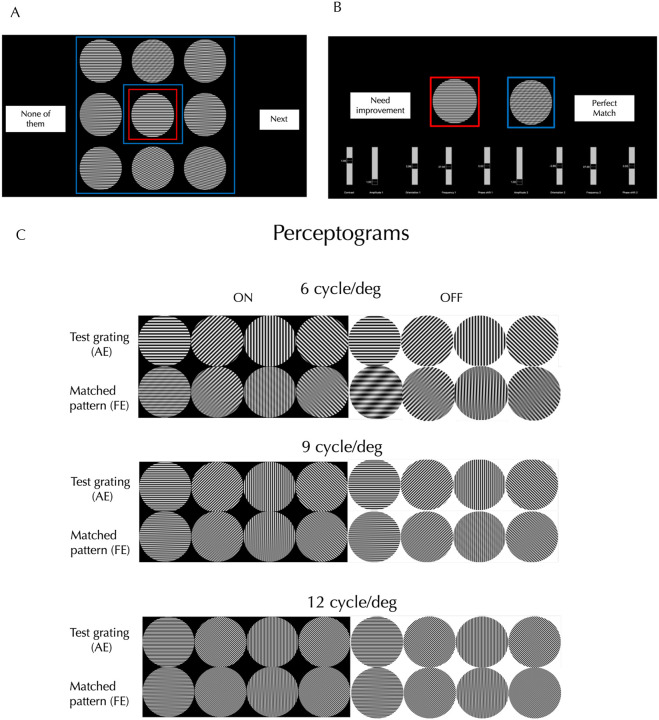
Perceptogram Procedure and Images: A. Initial Configuration: The amblyopic eye (AE) views a central single grating (red border), while the fellow eye (FE) sees a surrounding test grating and seven plaid patterns, each representing a different distortion type (blue borders). The observer selects the surrounding pattern that most closely matches the central grating. **B. Matching Procedure:** The observer fine-tunes four parameters of each of the two gratings within the selected plaid pattern seen by the FE to make a perceptual match to what the AE sees when shown the test grating. **C. Results:** Eight ON and OFF gratings per spatial frequency and the corresponding perceptograms are shown for Obs 3 for one session.

The accuracy of drawings depends on the observer’s skill and the tools provided, and could misrepresent contrast, shading, spatial frequency (SF), orientation (OR), duty cycle, or linearity, so for higher fidelity, we measured perceptograms for 5 amblyopes ages 22–45 using computer generated stimuli. The resemblance of the drawn forms to sinusoidal plaids simplified stimulus generation. Observers viewed a dichoptic display, consisting of two orthogonally polarized LCDs passed through a beam splitter (Supplemental Figure S2) and seen through glasses polarized orthogonally so that one screen is seen by the AE and the other screen by the FE. The AE was shown a 3 dva (degrees of visual angle) test grating in the center (6, 9 or 12 cyc/deg, 0, 45, 90 or 135° orientations, ON or OFF). Either simultaneously or in alternating sequence, the FE was shown eight 3 dva patterns surrounding the test: 7 plaids matching the different distortion types illustrated in Figure S1, adjusted to the SF and OR of the test, plus an exact copy of the test grating (Figure 1A). The observer picked the pattern configuration most like the central image, ignoring possible differences in contrast, SF, OR and phase and scotomas. If the observer chose the identical grating, the trial ended. Otherwise, the display showed the AE just the test grating and adjacent to it showed the FE just the chosen match (Figure 1B). The chosen plaid was modified to generate better matches by varying contrast, SF, OR and phase of each of the 2 component gratings, until the percept of the test grating seen by the AE was matched perfectly by the generated match in the FE. The procedure was repeated for each grating in three different sessions. Since the amblyopic distortions have been well documented with a large sample^7^, we concentrated on getting complete sets of perceptograms for the three amblyopes in our sample who saw distortions and deriving a model of amblyopic cortex for each observer that could generate all the images including the veridical percepts.

The 24 matched ON and OFF perceptograms for each observer were used together to infer cortical neural deficits in the ON and OFF systems. We assumed the following linking hypothesis for our model: When the two patterns presented dichoptically look identical to an observer, the signals generated by the test grating seen through AE at some stage of visual cortex match the signals generated by the perceptogram seen through FE. As the simplest instance, we further assumed that AE V1 filters are linear transforms of FE V1 filters. For our model, we separately passed the image of the grating and the perceptogram through orientation-selective Steerable Filters representing a normal V1 cortex and then directly derived linearly transformed filters that collectively generated signals from single gratings that matched signals from perceptograms generated by the unmodified filters. The receptive fields (RF) of the modified filters showed changes in spatial frequency and orientation selectivity but also more complex changes, and a marked increase in spatial extent. The transformed filters can provide a target for physiological measurements, neural development models of amblyopic cortex and for tailoring treatments to ameliorate functional problems caused by amblyopia.

The processing deficits that led to the form phantoms could be adequately explained just in terms of linearly transformed RFs, so we next aimed to explain deficits in other form judgments that would require combinations or correlations of multiple filters. A diagnostic case is judging deviations from a perfectly circular blurred contour when it is replaced with a sinusoidal contour^15^. For a set of fixed frequencies of the distorting waveform, the threshold amplitude for the AE and FE to judge the presence of sinusoidal deviations provides a probe of global form processing^16^. We show that the AE filters derived for our observers, in conjunction with a rotational correlation, predict the change in thresholds for both eyes as a function of modulation frequency, with higher thresholds for the AE, consistent with the published data.

The form phantoms suggest deficits of orientation processing, but there is no direct method to test our models by measuring RFs of single neurons in human visual cortex. A recent reverse correlation study showed broader perceptive orientation tuning for the AE than for eyes of normal observers for vertical gratings^17^, but our perceptograms show that some amblyopes see phantoms and some do not, and neither the phantoms nor the transformed filters are invariant to orientation. Consequently, for all our observers, we estimated perceptive fields^18^ for 0, 45, 90 or 135° orientations of ON and OFF gratings separately for AE and FE using reverse correlation psychophysics^19–20^. Our observers found the task difficult, leading to noisy results especially for the AE, and the procedure cannot identify the cortical locus of the modified filters, but the perceptive orientation tuning through AE was broader, weaker, and shifted compared to FE orientation tuning for the observers who saw distortions, but not for those who did not, providing additional insight into the orientation deficits underlying the form phantoms.

Note: A major component of this work is mathematical and computational. We explain the operations in words in the main text and present the mathematical details in the Supplemental Information under the same headings as the corresponding text sections. The Supplemental Figures include results for individual observers.

## RESULTS

### Perceptograms

Three out of our 5 amblyopic observers (Table 1) reported seeing form phantoms in gratings shown to the AE, 60% versus 67% in the much larger sample^7^. Since each observer’s images take up a lot of space, in the main text we present one observer’s perceptograms and models in detail and present the others in the Supplementary Information. Figure 1C shows the 24 gratings and their corresponding perceptograms from a single session by Obs 3, who saw the largest number of distortions. The left four columns display ON gratings, and the right four columns display OFF gratings: the same gratings are ON or OFF depending on being increments or decrements from a black or white background respectively. Each orientation-frequency combination produced a distinct phantom pattern. Stimulus polarity also influenced perception: for example, at 90° and 6 cycles/deg, the ON grating produced a net-like appearance, while the OFF grating yielded a smoother, wavy structure with shadows at the top and bottom, suggesting that deficits could be different for the ON and OFF systems.

Complete sets of perceptograms from all three observers who saw phantoms are shown in Supplemental Figure S3, excluding the 12 cycles/deg condition for Obs 4, who reported only a uniform gray disk due to the spatial frequency exceeding their resolution limit. Across observers, the most frequently reported percepts resembled a grating with one or more blurred segments separating phase-shifts, like those illustrated in rows 2–5 of Figure S1. These patterns emerged from the combination of two gratings with closely matched spatial frequencies and contrast, but with slight differences in orientation and phase. The second most common patterns were net-like, resulting from the combination of one grating like the test and another with a lower spatial frequency, reduced contrast, and a different orientation (top row Figure S1). The bottom two patterns arise when the two constituent gratings are quite different in orientation from the test grating, with an interrupted pattern if they are the same contrast, and a wavy pattern if one is of lower contrast. The Deep Image Structure and Texture Similarity (DISTS) metric^21^ quantified the estimated perceptual distance between the perceptograms and the test gratings averaged over all the conditions for the three observers who saw distortions as 0.204 and significantly different from 0.0 that denotes a perfect perceptual match. The average perceptual distances between observers, spatial frequencies, orientations and ON/OFF polarities were not significantly different (Figure S4 Top).

In only 16 out of the 216 cases (7.4%), the summation of two sinusoidal gratings failed to fully capture the percept. In these cases, observers verbally reported the mismatches—generally describing contrast mismatches. When contrast mismatches were insufficient descriptions, observers provided additional verbal descriptions (included in the far-right column of Figure S3), often reporting irregularities across the patterns. When verbal descriptions were inadequate, observers illustrated their percepts using Procreate on an iPad, after being trained to control linearity, width, contrast, shading and orientation to make these drawings much more representative of the percept than the previously published pencil drawings. A small number of drawings showed some waviness not captured by the gratings. A careful drawing could take more than 30 minutes, making it unfeasible to use that method for the complete set of stimuli.

Across sessions, perceptograms were largely consistent within observers, with most variations reflecting minor differences in phase alignment. However, occasional session-to- session variability in reported pattern type was observed. For example, at 0°, 6 cycles/deg, for the ON stimulus, Obs 3 reported a net-like pattern in two sessions but described a grating with a shadow in the third. The exact cause of this variability remains to be investigated, particularly fixation problems for the amblyopic eye.

The 92.6% of the distortions that were accepted as matched by the digitally generated plaids, along with the verbal descriptions and Procreate drawings of the rest, demonstrate the importance of measuring the perceptograms, because it seems that the lack of gradual shading in the published drawings leading to depictions as lines or square wave gratings, sometimes with unequal duty cycles, are due to limitations of drawing skills with pencil and paper and do not represent perceived distortions. In addition, unlike the incomplete set of published drawings, we have complete sets of perceptograms for each observer, allowing us to build models that simultaneously account for both form distortions and veridical percepts. Therefore, we build cortical models that reproduce perceptograms instead of the drawings. If the irregularities and waviness were frequent, we would have incorporated neural scrambling or under-sampling in the models, but the occurrences were too rare.

### Cortical model

**Figure 2.**
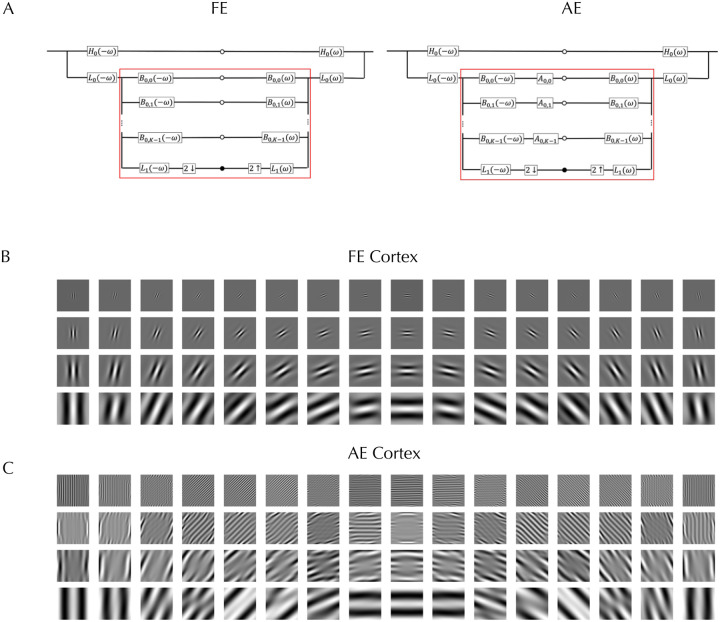
Cortical Model. A (FE). Standard Steerable Pyramid: The left side shows the complex steerable pyramid decomposition, and the right side shows the reconstruction process. The input image is split into high- and low-pass bands. The low-pass band is further decomposed into a lower-frequency band and a set of orientation sub-bands. White dots represent single sub-bands, while black dots denote sub-bands at the next coarser scale. The symbols ↓ and ↑ indicate downsampling and upsampling operations, respectively. The region outlined in red is repeated across a specified number of scales. **A (AE) Modified Pyramid:** A linear Amblyopic Transformation *A*_*i,k*_ is applied to each band-pass oriented filter. **B : Fellow Eye Filters:** Visualization of each orientation sub-band from the standard steerable pyramid (representing the FE cortex), showing the response to a central impulse. **C: Amblyopic Eye Filters:** Visualization of each orientation sub-band from the amblyopic steerable pyramid for the Obs 3. (representing the AE cortex), also in response to a central impulse.

We began with the working hypothesis that the form phantoms observed in amblyopia result from receptive field (RF) alterations in cells of early visual cortex that get input from the AE while the RFs are normal for cells that get input from the FE, with later visual areas processing the signals similarly irrespective of eye of origin. To model the responses of the FE driven normal V1 cortex to the stimuli, we used steerable pyramids for frequency decomposition of input images in the Fourier domain^22^. This method is translation- and rotation-invariant, prevents aliasing, and allows for perfect reconstruction of the original image. The block diagram in Figure 2A (FE) illustrates the standard architecture of the complex steerable pyramid, and the supplemental section gives the mathematical details. The input image is first decomposed into high-pass and low-pass components by multiplying its Fourier transform with a high-pass filter that emphasizes higher spatial frequencies, and a low-pass filter that retains lower frequencies. Both filters are selective only for spatial frequency and are independent of orientation. The low-pass band is subsequently decomposed into oriented bandpass filters. The process in Figure 2A is repeated across the specified number of scales. To visualize the spatial profile of these filters, a centered delta function was passed through each filter and the inverse Fourier transform applied. The V1 filters for the FE are shown in Figure 2B for 16 orientations and four spatial scales arranged from high frequency (top) to low (bottom), each with 16 orientations. The resulting patterns resemble oriented Gabor filters and by passing perceptograms through the complete set of filters, we ascertained that they satisfy perfect reconstruction.

Our linking hypothesis is that the signals sent to later stages of visual cortex of the test grating through AE early cortical neurons match the signals from the perceptogram through FE neurons. We tested if linear transforms applied to all standard oriented band-pass filters (Figure 2A AE) can collectively account for the cortical deficits, which allows us to write a formal equation equating the signals from the gratings through the linearly transformed filters to the signals from the perceptograms through the normal filters. The supplement shows that this equation can be simplified to directly derive the linear transformation from the ratio of the cross-spectrum to the power-spectrum. As an example, the transformed AE filters for Obs 3 for the ON condition are visualized in Figure 2C. Comparing Figures 2B and 2C shows that some of the AE RF distortions could be changes in spatial frequency and orientation tuning of V1 cells, corresponding to linear operations of scaling and rotation, but others seem to create complex non-Gabor RFs suggesting combined operations including shearing, particularly for diagonally oriented components. The transformed filters also exhibit an increase in effective spatial extent for the high frequencies as the ON and OFF lobes repeat over the complete 3 dva of the stimulus. The model cannot specify if these changes are in V1 RFs or in later combinations of V1 outputs. Similar transformations are evident in the complete set of ON and OFF filters for the three amblyopes who saw distortions (Supplemental Figure S5).

**Figure 3.**
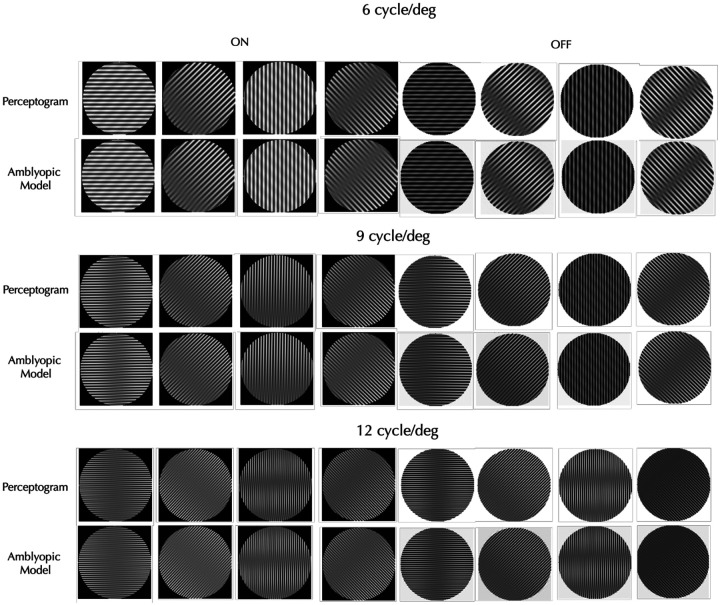
Perceptograms vs. Model Reconstructions. The left four panels correspond to ON stimuli, while the right four correspond to OFF stimuli. Each column represents a different stimulus orientation (the same stimulus sequence as Figure 1C). Within each stimulus pair, the top image shows the observer’s perceptogram, and the bottom image shows the corresponding reconstruction from the linearly transformed V1 filters representing the AE cortex. The model accurately generates the perceptograms.

We built models that covered all three spatial frequencies and four orientations, but separately for each observer, session, and stimulus type (ON vs. OFF). To test if each discrete set of linearly transformed filters is adequate, we applied the complete set in the Fourier domain (including the untransformed and unoriented high-pass and low-pass filters) to the Fourier transforms of the grating set, and then took the inverse Fourier transform to generate the corresponding perceptograms. An example comparison is shown in Figure 3 for the Obs 3 perceptograms from Figure 1C. Within each pair, the top image depicts the observer’s perceptogram, while the bottom shows the corresponding reconstruction from the AE model. Comparisons for all perceptograms for all observers are presented in Supplemental Figure S3. Visual inspection shows that each model accurately predicts the observer’s percepts with remarkable fidelity. The DISTS^21^ estimated perceptual distance between the model generated and measured perceptograms averaged over all the conditions for the three observers who saw distortions was 0.004 and not significantly different from 0.0 that denotes a perfect perceptual match. The average perceptual distances between observers, spatial frequencies, orientations and ON/OFF polarities were all not significantly different from 0.0 (Figure S4 Bottom).

### Sinusoidal Deformations from Circularity

**Figure 4.**
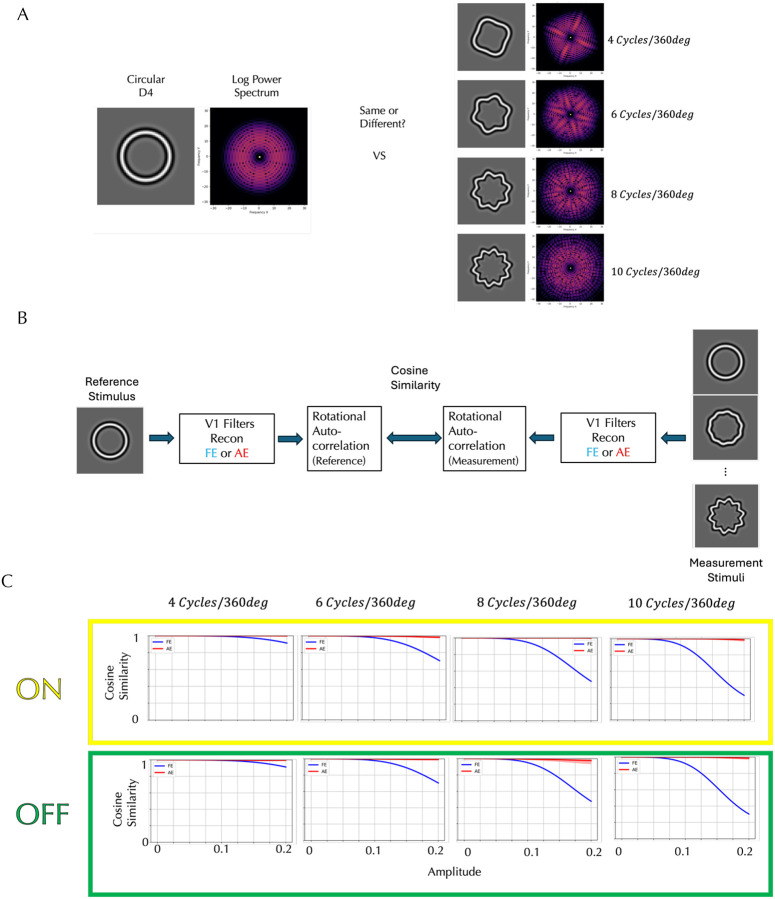
Detection of Sinusoidal Deformations from Circular D4 Stimuli and Model Simulations. **A:** Circular and deformed D4 stimuli and their log power spectra. While the circular D4 displays uniform power across orientations, the deformed D4s show localized increases in power at specific orientations, forming ridge-like structures. Amblyopic eyes consistently exhibited higher amplitude thresholds across radial frequencies than fellow eyes. **B:** Model for responses of the fellow eye and amblyopic eye. The statistical representation of the circular D4, processed through either the fellow eye (FE) or amblyopic eye (AE) V1 filters and then through a rotational correlation, is compared to those of the modulated D4 stimuli at various amplitudes using cosine similarity. **C:** The top row shows predictions from the ON amblyopic model (Yellow box), and the bottom row from the OFF amblyopic model (Green box), averaged over the observers who saw distortions (shading indicating ±1 standard deviation). Each row corresponds to a different radial frequency (4–10 cycles/360°). Each panel plots cosine similarity as a function of amplitude, where a higher similarity leads to a higher detection threshold. For the FE (black curves), cosine similarity decreases with increasing amplitude, and this reduction is more pronounced at higher radial frequencies. The AE curves (red) show relatively high cosine similarity across amplitudes and radial frequencies. Both are consistent with published results.

We tested our model on results of a task shown to have a global component: the detection of sinusoidal deviations from circularity of the fourth derivative of a Gaussian (D4)^16^. The left panel in Figure 4A shows the circular D4 stimulus and its log power spectrum, while the right panel shows one of four sinusoidally deformed D4s, each with a radial frequency of 4, 6, 8 or 10 cycles per 360° with its corresponding log power spectrum. The circular D4 exhibits uniform power across all orientations, whereas the sinusoidally modulated D4s reveal localized increases in power at specific orientations, producing the same number of ridge-like structures in the spectrum as the number of cycles per 360°. As modulation amplitude increases, the local differences from the circular D4 (far left) become more perceptually apparent, and the ridges in the power spectrum more pronounced. Detecting these orientation-specific enhancements is critical for identifying deformation, but there is also a curvature detection aspect to this task beyond judging local orientation deviations. When the circular and modulated D4 stimuli were presented simultaneously, and the detection threshold for the modulation was measured separately for the AE and FE^15^, detection thresholds for FE decreased with increasing radial frequency, but the AE thresholds were fairly constant and consistently higher than FE across all tested frequencies.

To investigate whether our AE V1 model can account for the observed difference in detection thresholds, we constructed a computational model with the schematic architecture shown in Figure 4B for the FE and AE simulations. In the fellow eye (FE) model, the circular D4 was processed through a simulated normal V1 pathway, and then we computed rotational correlations of the reconstructed image derived from the filter responses for rotation angles from −17° to 17° in 1° increments, yielding a feature vector of length 35. Feature vectors were computed similarly for modulated D4 stimuli with varying amplitudes and compared to the reference vector using a cosine similarity metric. In the amblyopic eye (AE) model, the same procedures were applied, but the stimuli were processed through the AE cortical model. This approach allowed us to quantitatively assess how closely the modulated stimuli resembled the circular reference under each cortical model.

The model predictions are presented in Figure 4C. Each panel represents a different radial frequency and compares the ON and OFF AE models to the FE models. Within each panel, cosine similarity is plotted as a function of modulation amplitude. Higher cosine similarity indicates greater representational overlap between the modulated and reference stimuli, which predicts a higher detection threshold. In the FE cortex model, cosine similarity systematically decreases as the amplitude of modulation increases, and the rate of decrease is greater at higher radial frequencies, reflecting that cortical responses show increased sensitivity to higher-frequency deformations. These findings are consistent with the psychophysical results which demonstrated lower detection thresholds for higher-frequency distortions. In contrast, the AE cortex model exhibits relatively high cosine similarity between the modulated and reference D4 stimuli across all amplitudes and radial frequencies. This pattern is consistent with a diminished sensitivity to form deviations in the amblyopic cortex, consistent with the higher and constant thresholds reported for AE. This insensitivity is observed in both the ON and OFF models of the AE cortex, although the OFF model tends to produce slightly lower cosine similarity values than the ON model, indicating a modest difference in form perception between the two pathways.

### Orientation Tuning via Reverse Correlation

**Figure 5.**
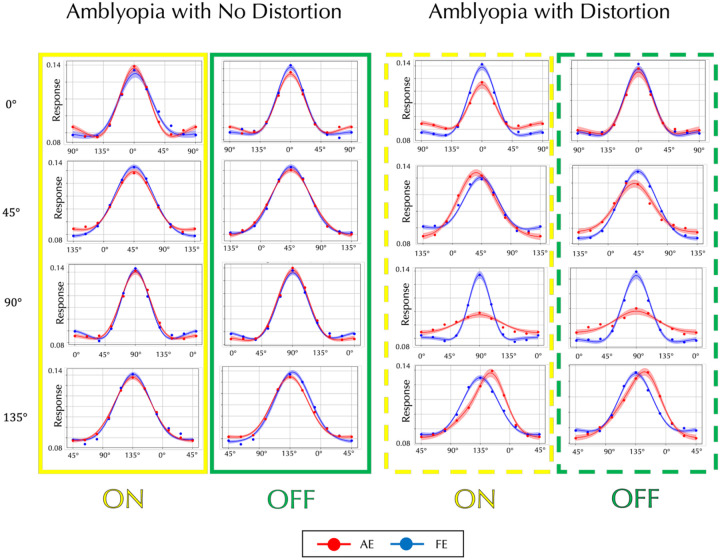
Perceptual Orientation Tuning Curves for Non-Distortion vs. Distortion Groups. The left two columns show the average results for amblyopic participants who did **not** report form distortions (Solid boxes Yellow: ON, Green: OFF). The right two columns (Dashed boxes) show results for those who **did** report distortions. Each row corresponds to a different target orientation. Within each panel, response magnitude is plotted against physical orientation. Response magnitude is scaled to give unit area under the curve. Red and blue dots represent the mean responses (after 5,000 bootstrap resamples) for the amblyopic and fellow eye conditions, respectively. Solid traces are the best fitting von Mises functions, with shaded areas indicating ±1 standard deviation. Most of the mean R-squared values were 0.98 or higher.

The AE filters for amblyopes who see form phantoms show complex differences from the FE filters, but the perceptograms and D4 results clearly point to deficits in orientation processing. To understand differences in orientation tuning between amblyopes who see phantoms and amblyopes who do not, we measured perceptive fields using reverse correlation (RC)^18–20^ for target gratings of 6 cyc/deg with 0°, 45°, 90°, or 135° orientation for ON and OFF conditions, separately for AE and FE (see Methods: Reverse Correlation). Participants fixated on the central display as a series of orientations were flashed and were instructed to press a button as soon as they detected the target orientation that had been shown to the FE. Given the difficulty that our amblyopic observers had doing the task, we used presentations of 100 msec, which is slower than usual. The supplemental section describes in detail how we obtained the orientation tuning curves shown in Figure 5. The left two columns (solid outlines; red for ON, blue for OFF) depict averaged responses from amblyopic participants who did not report form distortions (Non-Distortion Group). The right two columns (dashed outlines) show data from participants who reported perceiving distortions (Distortion Group). Each row corresponds to a different target orientation, and within each panel, response magnitude is plotted against the physical orientation of the stimulus. Red and blue dots represent the mean responses (based on 5,000 bootstrap resamples) for the amblyopic and fellow eyes, respectively. Solid curves denote the best fitting von Mises functions, with shaded bands indicating ±1 standard deviation. In the Non-Distortion Group, amblyopic and fellow eye tuning curves are roughly similar, but the Distortion Group clearly shows that the AE has reduced peak responses at 0° and 90°, broader tuning at 90°, and marked peak shifts at 45° and 135°. There are some differences between ON and OFF results as the ON curves are slightly broader, but they do not provide sufficient evidence that either system is more affected. Tuning curves for individual observers are plotted in Supplemental Figures S5 and S6.

## DISCUSSION

This paper solves a 20-year-old puzzle which has great significance for understanding amblyopic vision, and designing potential treatments, but the most significant conclusion of this work is that changes in neuronal receptive fields have profound effects on perception, to the extent that observers can see more features than are present in the viewed stimulus. The phantoms/distortions persist despite amblyopes having years to potentially adapt to the transformed RFs. Receptive fields in normal visual cortex seem to be exquisitely tuned for perception to be veridical in most cases.

The efficiency of orientation processing has been linked to the anisotropy in the distribution of image orientations in natural scenes being matched by the distribution of orientation tuned cells in V1^23^. The less-than-optimal consequence is that the anisotropy in the distribution of orientation and direction tuned cells, leads to anisotropy in the estimates of angles^9^, orientation flows^9^ and optic flows, which makes high level percepts of 3D shapes^9^ and object non-rigidity^24^ orientation-dependent, but leaves forms recognizable. Distortions of receptive fields seem to be more disruptive of perception than variations in distributions of neuronal properties.

Amblyopia is clinically identified by a reduction in the visual acuity of the AE and disrupted binocular function, and these have been the focus of most research, but deficiencies in several low- and high-level perceptual abilities have also been documented, although not adequately understood in terms of neural substrates or developmental mechanisms. We definitively confirmed the published perceptual phantoms with perceptograms and showed that they can be explained by linear transformations of V1 RFs. Many transformed RFs resembled V1 RFs albeit with broader tuning and shifted peak, but others were much more complex and may reflect combinations of V1 outputs at later stages. Many of the linearly transformed filters in Figure 2C have many more lobes than the normal filters, which gives greater summation area for each RF and narrower tuning. This RF structure implies that the gratings that fill the whole RF while matching spatial frequency selectivity would evoke the maximum responses, unlike for normal RFs, which could partially explain the much larger increase in contrast sensitivity for amblyopes as compared to normals as field sizes are increased from 0.25 dva to 1.0 dva^25^, although the greater increase up to field sizes of 8 dva would probably be due to enhanced lateral connections. The neural development of such abnormal RFs requires a larger spatial extent of inputs, whether it is thalamic inputs to V1 or V1 inputs to V2. Visual blur effectively reduces stimulus contrast, which leads to lower neuronal responses but increased cortical spread^26^. During the amblyopic critical period, blur has been shown to reduce neuronal contrast sensitivity^27^, but could also affect the formation of connections, leading to larger RFs or extended lateral connections.

The formal equation between the AE response to gratings and the FE/V1 response to their matched perceptograms, enabled us to derive the transformed AE filters, but does not address their cortical loci. In principle the equation could be used to linearly transform a set of normal RFs in any cortical area. If V2 RFs were as well established as V1, we could have also applied the equation to V2, especially since amblyopia has been shown to disrupt RF structure in V2 neurons^28,29^ possibly even more than it does in V1^27^. However, while it is known that V2 RFs are weighted combinations of large numbers of orientation tuned V1 RFs, and thus have much more intricate internal structure^30^, which corresponds to heightened sensitivity to combinations of discontinuous orientations^31^ and textures^32^, there is no established canonical form that we could have used in the model.

The transformed AE RFs were not sufficient to explain the reduced sensitivity to the global judgement involving sinusoidal perturbations of contours, but required the addition of rotational correlation, which is more general than a model involving curvature specific neuronal connections^33^. Computational models of V2 neuronal sensitivities to naturalistic textures include many different correlations between V1 outputs^23,32^, and it is likely that some V4 neurons that are sensitive to concentric stimuli^34^ incorporate rotational correlations between earlier outputs. The perceptual phantoms in the perceptograms involve oriented gratings which suggest deficiencies of orientation processing. Consistent with this idea, the results of the reverse correlation experiment showed orientation tuning differences between AE and FE for amblyopes who saw phantoms, but not for those that did not.

Amblyopia is generally thought to result from relative monocular deprivation of one eye causing a shift in ocular dominance of binocular neurons in V1 during an early critical period^35,36)^. However, multiple instances of abnormalities in areas beyond V1^28,29^ require more than a shift in ocular dominance^37^. Our results suggest that neural development of distortions in RFs need to be further investigated as a consequence of amblyopia. Our initial conjecture on starting this project was that the development of frequency and orientation selective V1 RFs requires balancing ON and OFF thalamic inputs^38^, so if they are not balanced, RFs will not develop normally. We had also found some evidence that the ON system is affected more than the OFF in amblyopia, as measured by grating resolution and salience of whites versus blacks in random checkerboards^39^, buttressing our idea. That provided the motivation to use separate ON and OFF gratings, but the perceptograms showed only a slightly stronger effect of amblyopia for ON versus OFF stimuli, and the effects were mixed in the reverse correlation results. In the perceptogram measurements, the stimuli were seen for prolonged periods with eye movements and may not have isolated ON and OFF systems very well, but the reverse correlation stimuli were flashed briefly, so they truly are increments and decrements. In addition, a comparison of the AE filters to FE filters in Figure 2 reveals that whereas some RFs do become less selective in the AE, the transformation in other RFs is better described as rearrangements of segments and is not due solely to a weaker ON or OFF system. Our results thus do not provide strong support for our initial conjecture, and the neural development of abnormal RFs remains an open question, with broad effects of reduced contrast signals during the critical period being an important candidate.

It is worth considering the functional implications of our results. Stereo is compromised in amblyopia, so amblyopes have to rely on monocular cues. However, many monocular cues are critically based on orientation information, so if oriented patterns are perceived as distorted the brain may not be able to compensate. There is strong evidence for orientation information being critical for 3D shape from texture^40–42^, mirror symmetry^43,44^ and 3D pose perception^45^. Figure S8 shows that whereas the diagnostic orientation flows in a 3D shape from texture stimulus^41^ are retained by the FE cortical model, they are lost by the AE model. Simulations using mirror symmetric complex images^43^ showed loss of detail by the AE model and lack of symmetry in the output for some but not all images. Our models predict that the amblyopic eye will show greater deficiencies in these functions. It is possible that if the AE models were derived from a more diverse sample of perceptograms, the filters may extract more information from such images but investigating performance on these tasks for amblyopic observers that do and do not see form phantoms would be worthwhile, as it could also test whether the non-amblyopic eye is sufficient.

It took a combination of perceptual perceptogram measurements and generative computational modeling to reveal how neuronal RF properties lead to veridical and phantom percepts. Invasive methods for measuring fine-grained neuronal properties such as electrophysiology and 2-photon imaging cannot be done in humans, and it remains to be seen if recording and perceptography^3^ in non-human primates can be expanded to a large enough scale. Non-invasive techniques such as fMRI and VEP would have to improve considerably in spatial and temporal resolution to answer such questions. For the foreseeable future Generative Phenomenology could be a powerful strategy to understand the neural bases of human perception.

## EQUIPMENT AND METHODS

### Observers

Five female observers with amblyopia participated in the study. Each had a prior clinical diagnosis of amblyopia and received a thorough optometric evaluation by the authors J.W. and B.R. prior to the experiment. Of the group, two had strabismic amblyopia and three had anisometropic amblyopia. Detailed participant data are presented in Table 1. All participants were unaware of the study's purpose and wore their best optical correction for the testing distance during the experiments. All participants provided written informed consent, and the study protocol was approved by the Institutional Review Board at the SUNY College of Optometry, in accordance with the tenets of the Declaration of Helsinki.

### Perceptograms

#### Apparatus

Figures 2A and 2B show the visual stimuli — test stimulus outlined in red and measuring stimuli outlined in blue — as presented using a Planar SD2620W Stereo/3D system. This setup consists of two calibrated LCD monitors positioned at right angles and optically combined using a beam splitter (see Figure S2). Each LCD monitor emits plane-polarized light, and the two monitor’s polarization axes were mutually orthogonal. The image in the beam splitter is viewed through glasses polarized so each eye receives input from only one monitor: the amblyopic eye (AE) views the test stimulus, and the fellow eye (FE) views the measuring stimuli. Prior to the experiment, we confirmed the absence of crosstalk between the two eyes and ensured that all nine patterns appeared evenly spaced. For one participant (Obs 4) with strong interocular suppression, the test and motion stimuli were alternated at a rate set by the observer. Each eye received a display resolution of 1920 × 1200 pixels at a 60 Hz refresh rate. Stimuli were presented and the experiment was controlled using PsychoPy, and all data were analyzed using Python. Head position was stabilized with a chin rest at a viewing distance of 1.2 meters. All experiments were conducted in a darkened room.

#### Stimulus generation

Using Python, we generated 24 test gratings that varied in orientation (0°, 45°, 90°, and 135°), spatial frequency (6, 9, and 12 cycles/deg), and stimulus polarity (ON: black background producing bright bars; OFF: white background producing dark bars). Each stimulus subtended 3° of visual angle. Phantom patterns were created by summing pairs of gratings, as illustrated in Figure 1.

#### Psychophysical procedures

At the start of the experiment, we confirmed that all the participants could perceive a veridical grating through the fellow eye (FE) by asking them to verbally describe the single grating. In the main task, the amblyopic eye (AE) viewed a central single grating (red outline), while the FE was presented with a surrounding test grating and seven plaid patterns, each representing a different phantom type (blue outlines), as shown in Figure 1A. The observer selected the surrounding pattern that most closely matched the central grating. As illustrated in Figure 1B, they then fine-tuned four parameters each of the component gratings of the selected plaid to improve the perceptual match. After adjustment, the observer judged whether the single grating viewed through the AE perceptually matched the plaid viewed through the FE. Gratings of the 4 orientations, 2 stimulus polarities, and 3 spatial frequencies, were presented in randomized order. Each session lasted approximately one hour, and the experiment was repeated on three separate days. If the perceptogram did not provide a perfect match, the observer first provided a verbal description of the mismatch. When verbal reports were insufficient, the observer sketched the perceived phantom using Procreate on a 12.9-inch iPad. The drawing was projected onto the Planar display seen by the FE, while the AE continued viewing the single grating.

### Reverse Correlation

#### Stimulus generation

To measure orientation tuning, we presented a rapid sequence of sinusoidal gratings randomly varying in orientation at 10 Hz, with each grating displayed for 100 ms. This temporal frequency was chosen for task feasibility, as higher frequencies were too difficult for some participants with amblyopia. Gratings had a spatial frequency of 6.0 cycles/deg—the lowest frequency used in the Perceptogram experiment—and were shown at full contrast. Stimuli were presented on the front-facing monitor of the Planar Display, without the beam splitter, centrally through a 3° diameter circular aperture, against either a white or black background. Each condition consisted of one of four target orientations (0°, 45°, 90°, or 135°) combined with one of the two stimulus polarities (ON or OFF), and was tested separately for the fellow and amblyopic eyes, yielding a total of 16 unique conditions. Each trial within a condition consisted of 600 gratings presented over 60 seconds. Gratings were randomly selected from 10 possible orientations (spaced 18° apart) and one of four phases (0, π/2, π, or 3π/2).

#### Psychophysical procedures

The experimental procedure followed protocols established in previous studies^17–20^. To assess each eye independently, participants wore an eye patch over the non-tested eye. Each trial consisted of a 1-minute sequence during which participants were instructed to press a button as quickly as possible upon detecting a grating with a specific orientation (horizontal, vertical, or oblique) embedded within the sequence. Trials began with brief on-screen instructions, followed by a brief tone signaling the onset of the grating sequence. Participants initiated each trial with a button press and were instructed to maintain central fixation throughout. After each trial, the instruction screen reappeared, allowing participants to rest before continuing. No performance feedback was provided. Each condition included 30 trials, lasting approximately 45 minutes in total. The full set of conditions—four orientations, two stimulus polarities (ON and OFF), and two eyes (fellow and amblyopic)—was administered in randomized order, typically with two conditions completed per day.

## Figures and Tables

**Table 1.** Observer details.

Subject	Age	Gender	Type of amblyopia	Visual Acuity(LogMAR)	Refraction	Fixation	Distortion
Obs 1	24	F	Anisometropic	OD: 0.00 OS: 0.20	OD: +6.75/−0.25 × 125 OS:+7.75/−1.00 × 075	Central	No
Obs 2	49	F	Combined	OD:0.20 OS:0.00	OD: +5.50 OS: +5.00	Central	No
Obs 3	22	F	Anisometropic	OD:0.00 OS: 0.30	OD: −2.50/−1.00 × 180 OS: +0.75/−1.00 × 180	Central	Yes
Obs 4	22	F	Combined	OD: 1.00 OS:0.00	OD: −22.00/−2.00 × 035 OS: −11.50/−2.75 × 140	Temporal	Yes
Obs 5	23	F	Anisometropic	OD: 0.40 OS: −0.10	OD: 0.00/−0.50 × 018 OS: −3.25/−0.50 × 121	Central	Yes

## Data Availability

All data and computational codes are available from the authors on request.

## SIGNIFICANCE STATEMENT

What we see is generated by our cortical neurons processing sensory input. With their amblyopic eye, whose signals are processed by deficiently developed cortical cells, many amblyopes see phantom forms that contain more features than the viewed stimulus, providing a perfect case for studying the neural generation of percepts (Generative Phenomenolog)y). We acquired computer aided perceptograms (viewable records of perceived images) of the phantom forms and then derived a cortical model that accurately generated the perceptograms by using linear transforms of normal V1 receptive fields. The modifications also explained amblyopic deficits at discerning departures from circularity and were validated with reverse-correlation experiments. These results show that small changes in cortical neuronal properties can cause major differences in what people perceive.
