# Supplementary material for: Phantom Forms in Amblyopic Vision and what they reveal about the Generative Brain
Source: bioRxiv. 2025 Nov 7:2025.06.24.661078. Preprint. [Version 4] doi: 10.1101/2025.06.24.661078 (PMC12262670; doi:10.1101/2025.06.24.661078)
Supplement: Supplement 2 [file NIHPP2025.06.24.661078v4-supplement-2.pdf]

bioRxiv preprint doi: https://doi.org/10.1101/2025.06.24.661078; this version posted November 7, 2025. The copyright holder for this preprint (which was not certified by peer review) is the author/funder, who has granted bioRxiv a license to display the preprint in perpetuity. It is made available under aCC-BY 4.0 International license.

Mathematical & Statistical Details

Cortical model

In this section, we provide the equations that describe how we built filters for FE and AE, and how we tested the adequacy of the filters to reconstruct the perceptograms. The FE filters are standard, but we describe details to be able to specify what was transformed for AE. Because the fellow eye (FE) did not exhibit any distortions, we assume that signals are processed like normal V1, and we model it with a steerable pyramid for frequency decomposition. The Steerable Pyramid python library is publicly available at https://docs.plenoptic.org/docs/branch/main/tutorials/models/Steerable_Pyramid.html .The steerable pyramid decomposition operates in the Fourier domain. Let $I(x, y)$ be the input image. Its 2D Fourier transform is $\mathcal{F}(I(x, y))$, and the inverse Fourier transform $\mathcal{F}^{-1}(\mathcal{F}(I(x, y)))$ reconstructs the original image from its frequency representation. Figure 2A illustrates the architecture of the complex steerable pyramid. The left side of the block diagram depicts the decomposition process, while the right side illustrates reconstruction. The decomposition is performed in polar coordinates, where the radial component r represents spatial frequency and the angular component θ corresponds to orientation. The high-pass and low-pass filters used in this transform are defined as follows:

$$L(r) = \begin{cases} 1 \quad if \;\; r \leq \frac{\pi}{4} \\ \cos\left(\frac{\pi}{2} log_2\left(\frac{4r}{\pi}\right)\right) \;\; if \;\; \frac{\pi}{4} \leq r \leq \frac{\pi}{2} \\ 0 \quad if \; r \geq \frac{\pi}{2} \end{cases} \qquad \#(S1)$$

$$H(r) = \begin{cases} 0 \quad if \;\; r \leq \frac{\pi}{4} \\ \cos\left(\frac{\pi}{2} log_2\left(\frac{2r}{\pi}\right)\right) \;\; if \;\; \frac{\pi}{4} \leq r \leq \frac{\pi}{2} \\ 1 \quad if \; r \geq \frac{\pi}{2} \end{cases} \qquad \#(S2)$$

bioRxiv preprint doi: https://doi.org/10.1101/2025.06.24.661078; this version posted November 7, 2025. The copyright holder for this preprint (which was not certified by peer review) is the author/funder, who has granted bioRxiv a license to display the preprint in perpetuity. It is made available under aCC-BY 4.0 International license.

The input image is first decomposed into high-pass and low-pass components by multiplying its Fourier transform with the filters, H_0 and L_0:

$$H_0(r) = H\left(\frac{r}{2}\right) \#(S3)$$

$$L_0(r) = L\left(\frac{r}{2}\right) \#(S4)$$

The low-pass band is subsequently decomposed into a lower-frequency band and a set of orientation-selective sub-bands through the combination of a high-pass filter and an angular mask ($G_k(\theta)$):

$$G_k(\theta) = \begin{cases} a_k\left[\cos\left(\theta - \frac{\pi k}{K}\right)\right]^{K-1}, if \ \left|\theta - \frac{\pi k}{K}\right| < \frac{\pi}{2} \\ 0, otherwise \end{cases} \#(S5)$$

Here, K is the total number of orientation-selective filters, k indicates the preferred orientation, and a_k is a normalization constant.

The orientation symmetric filer in the first scale, $B_{0,k}$ is expressed as:

$$B_{0,k} = H_1(r)G_k(\theta) = H(r)G_k(\theta)\#(S6)$$

Following this cascade, L_0 is first applied to isolate the low-frequency band, and then multiplied by $B_{0,k}$ to create an oriented band-pass filter that is selective for both spatial frequency and orientation. To proceed to the next scale (i.e., lower spatial frequency), the model applies $L_1(= L)$ and repeats the process by multiplying it with the orientation-symmetric filter $B_{1,k}$ $(= H_2G_k = H(2r)G_k)$. The filter response is zero beyond $|r|>\pi/2$, allowing computations to be limited to $|r|<\pi/2$ for efficiency. When filters across scales are combined, they are zero-padded to ensure matching dimensions before summation. This process is repeated across the specified number of scales.

In general, the orientation-symmetric filter at the i-th scale and k-th orientation, denoted $B_{i,k}$, is defined as:

$$B_{i,k} = H_{i+1}G_k = H(2^i r)G_k(\theta)\#(S7)$$

i-th scale lowpass filter $L_i(r)$ is $L(2^{i-1}r)$. Therefore, the oriented band-pass filter at the n-th scale and k-th orientation is constructed as:

$$Q_{n,k} = \left(\prod_{i=0}^{n} L_i\right)B_{n,k} \ \#(S8)$$

bioRxiv preprint doi: https://doi.org/10.1101/2025.06.24.661078; this version posted November 7, 2025. The copyright holder for this preprint (which was not certified by peer review) is the author/funder, who has granted bioRxiv a license to display the preprint in perpetuity. It is made available under aCC-BY 4.0 International license.

To visualize the spatial profile of these filters, a centered delta function $\delta(x,y)$ is passed through each filter. The resulting spatial domain representation is obtained by:

$$\mathcal{F}^{-1}\left(\left|Q_{n,k}\right|^2 \mathcal{F}\big(\delta(x,y)\big)\right) \#(S9)$$

These visualizations are shown in Figure 2A, where four spatial scales are arranged from top to bottom (from high to low frequency), each with 16 orientations. The responses of these filters satisfy perfect reconstruction under the L^2-norm.

Figure S4B illustrates the architecture of the complex steerable pyramid for AE incorporating the Amblyopic linear transformation $A_{i,k}$ for each i-th scale and k-th orientation filter. The model follows the same steps as in the FE V1 pathway, except that the output of each $B_{i,k}$ is transformed by$A_{i,k}$. We estimated $A_{i,k}$ as follows: Let $\boldsymbol{S}$ ($\in R^{HW\times C}$) be a matrix containing vectorized single grating stimuli, where H and W are the image height and width, and C is the number of conditions. Similarly, let D ($\in R^{HW\times C}$) represent the corresponding perceptograms. We assume the oriented band-pass filter response $Q_{i,k}$ is subjected to a linear transform $A_{i,k}$ ($\in R^{HW\times HW}$), yielding the AE cortical response:

$$Y_{A_{i,k}} = A_{i,k} diag\left(vec\big(Q_{i,k}\big)\right)\mathcal{F}(\boldsymbol{S}) + N_{A_{i,k}} = A_{i,k}X_{i,k} + N_{A_{i,k}} \#(S10)$$

Where $N_{A_{i,k}}$ represents an independent noise source and $X_{i,k} = diag\left(vec\big(Q_{i,k}\big)\right)\mathcal{F}(\boldsymbol{S})$. The corresponding FE response to the perceptograms is:

$$Y_{F_{i,k}} = diag\left(vec\big(Q_{i,k}\big)\right)\mathcal{F}(\boldsymbol{D}) \#(S11)$$

Assuming the linking hypothesis that $Y_{A_{i,k}} = Y_{F_{i,k}}$, the cross-spectrum between $Y_{F_{i,k}}$ and $X_{i,k}$ becomes:

$$P_{YX} =< Y_{F_{i,k}} X_{i,k}^* >$$

$$=< Y_{A_{i,k}} X_{i,k}^* >$$

$$= A_{i,k} P_X \#(S12)$$

Where $X_{i,k}^*$ is the conjugate transpose of $X_{i,k}$ and $P_X =< X_{i,k} X_{i,k}^* >$. Then, the amblyopic transformation $A_{i,k}$ can be estimated as:

$$A_{i,k} = P_{YX} P_X^{-1} \#(S13)$$

bioRxiv preprint doi: https://doi.org/10.1101/2025.06.24.661078; this version posted November 7, 2025. The copyright holder for this preprint (which was not certified by peer review) is the author/funder, who has granted bioRxiv a license to display the preprint in perpetuity. It is made available under aCC-BY 4.0 International license.

The AE V1 filters for Obs 3 for the ON condition are visualized in Figure 2B using the same method as Equation S9.

For each observer, session, and stimulus type (ON vs. OFF), we tested whether the discrete set of filters for that condition could adequately reconstruct the perceptograms. For $S(x, y)$ a grating from the stimulus set, and $D(x, y)$ the corresponding perceptogram, we passed the Fourier transform of the grating through the appropriate set of AE filters, and then compared the inverse Fourier transform which gave the reconstruction to the perceptogram:

$$\mathcal{F}^{-1}\left\{\left(|H_0|^2 + \sum_{i=0}^{N-1}\sum_{k=0}^{K-1}\left|A_{i,k}Q_{i,k}\right|^2 + \prod_{i=0}^{N}|L_i|^2\right)\mathcal{F}\big(S(x,y)\big)\right\} \approx D(x,y) \quad \#(S14)$$

Sinusoidal Deformations from Circularity

This section describes the autocorrelations computed for the simulations. Individuals with amblyopia exhibit a deficit in detecting sinusoidal modulations from circularity of the fourth derivative of a Gaussian (D4)[15]. Using similar procedures as above, we computed FE and AE filter responses for the D4 reference and various amplitudes for each frequency of modulation. The image was reconstructed using Equation S14, followed by computation of its rotational correlation. Each rotational autocorrelation was computed as:

$$R(\theta) = \int_{\mathbb{R}^2} U(x,y)U_\theta(x,y)dxdy \quad \#(S15)$$

$U(x, y)$ is the reconstructed image by the methods above and U_θ is $U(x, y)$ rotated by angle θ around the origin. We sampled rotation angles from $-17°$ to $17°$ in $1°$ increments, yielding a vector of length 35. Cosine similarity between the auto-correlation vectors of the reference and modulated shapes was computed to generate the plots shown in Figure 4C.

Orientation Tuning via Reverse Correlation

This section provides details of the data analysis of the reverse correlation experiment. In the Reverse Correlation Experiment, observers fixated on the central display and were instructed to press a button as soon as they detected the target orientation. Grating orientations presented during the 1-second window (10 frames) prior to each key press were collected and used to construct a histogram of occurrences across time (τ) and orientation (θ). The concatenated histograms were combined into a heatmap as a function

bioRxiv preprint doi: https://doi.org/10.1101/2025.06.24.661078; this version posted November 7, 2025. The copyright holder for this preprint (which was not certified by peer review) is the author/funder, who has granted bioRxiv a license to display the preprint in perpetuity. It is made available under aCC-BY 4.0 International license.

of orientation (θ) and time (τ). Each trial consisted of a 600-frame (60-second) time series, which was resampled 5,000 times using bootstrap resampling. To reduce noise, the heatmap was smoothed using a small symmetric Gaussian kernel with $\sigma_\theta = 6°$ and $\sigma_\tau = 0.033$ seconds. The resulting heatmap was then normalized to produce PF(θ,τ). Individual PF(θ,τ) were computed for the Non-Distortion and Distortion Groups. As the individual trends within each group were consistent, we averaged the data across two observers for the Non-Distortion Group and three observers for the Distortion Group. To examine orientation tuning more closely, we marginalized the PF(θ,τ) over τ to obtain a tuning curve. The resulting data were then fit with an additive von Mises function:

$$PF_{fit}(\theta) = \alpha E(\theta) + \beta I(\theta) + c \#(S16)$$

$E(\theta)$ and $I(\theta)$ denote the excitatory and inhibitory components of the orientation tuning curve, respectively. The parameters α, β, and c modulate the amplitudes of excitation and inhibition, as well as the baseline level of the response. Both $E(\theta)$ and $I(\theta)$ are defined using normalized von Mises functions:

$$E(\theta) = \frac{\exp\big(\kappa_E \cos\big(2(\theta - \theta_E)\big)\big) - \exp(-\kappa_E)}{\exp(\kappa_E) - \exp(-\kappa_E)} \#(S17a)$$

$$I(\theta) = \frac{\exp\big(\kappa_I \cos\big(2(\theta - \theta_I)\big)\big) - \exp(-\kappa_I)}{\exp(\kappa_I) - \exp(-\kappa_I)} \#(S17b)$$

Here, κ_E and κ_I determine the tuning widths of the excitatory and inhibitory components, while θ_E and θ_I represent their respective preferred orientations.
