# Supplementary material for: Generative Phenomenology of form perception: Perceptograms and cortical models for amblyopic phantom percepts
Source: bioRxiv. 2025 Aug 19:2025.06.24.661078. Preprint. [Version 3] doi: 10.1101/2025.06.24.661078 (PMC12262670; doi:10.1101/2025.06.24.661078)
Supplement: Supplementary file 1 [file NIHPP2025.06.24.661078V3-supplement-1.pdf]

bioRxiv preprint doi: https://doi.org/10.1101/2025.06.24.661078; this version posted August 19, 2025. The copyright holder for this preprint (which was not certified by peer review) is the author/funder, who has granted bioRxiv a license to display the preprint in perpetuity. It is made available under aCC-BY 4.0 International license.

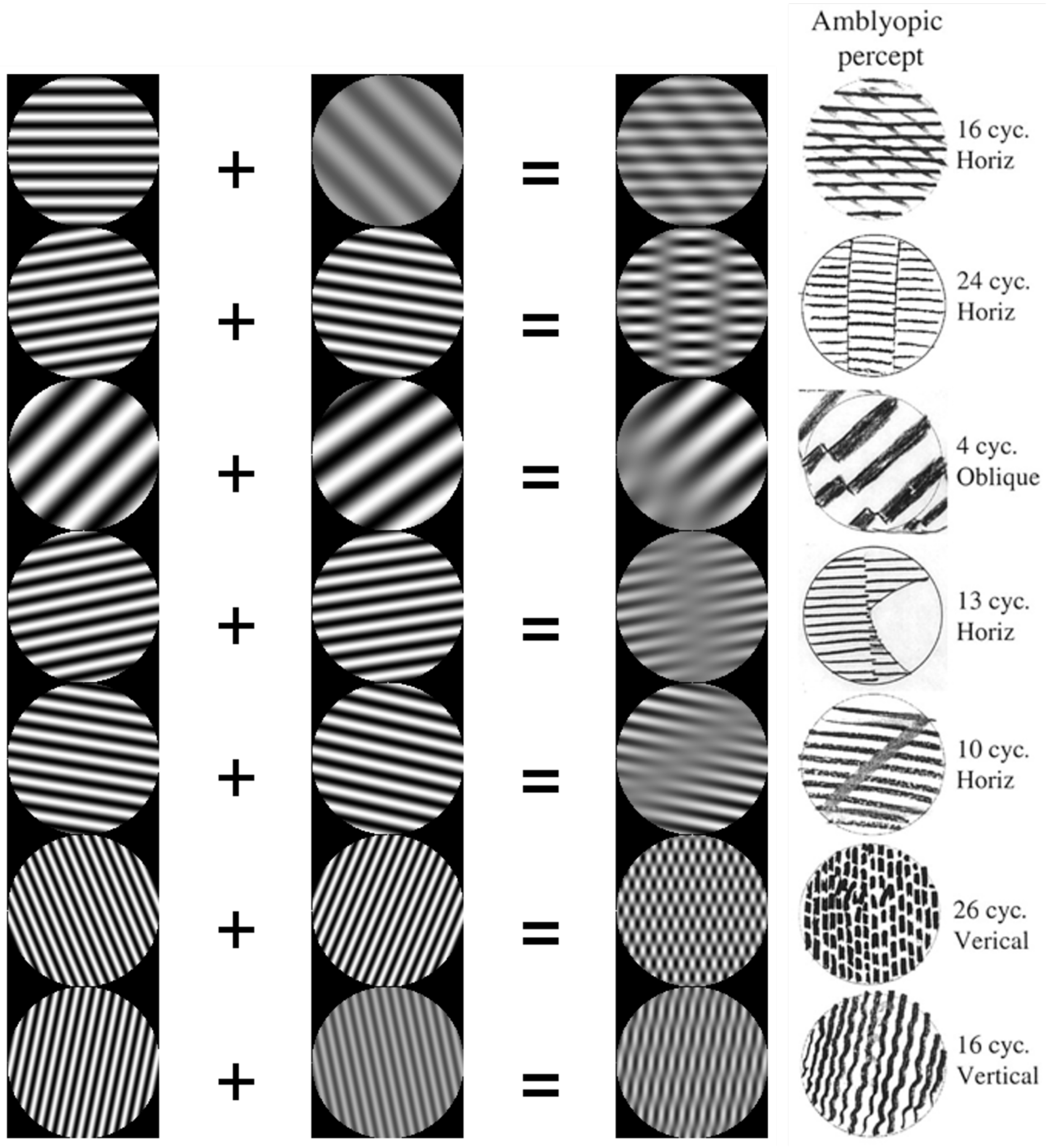

Figure E1. Drawings made by amblyopes matched by plaids consisting of sums of gratings:
Right: Drawings of seven classes of phantom percepts evoked by the sinusoidal gratings described by # cycles and orientation. Middle: Plaids resembling the drawn phantoms, produced by sums of two component gratings. Left: Component gratings.

bioRxiv preprint doi: https://doi.org/10.1101/2025.06.24.661078; this version posted August 19, 2025. The copyright holder for this preprint (which was not certified by peer review) is the author/funder, who has granted bioRxiv a license to display the preprint in perpetuity. It is made available under aCC-BY 4.0 International license.

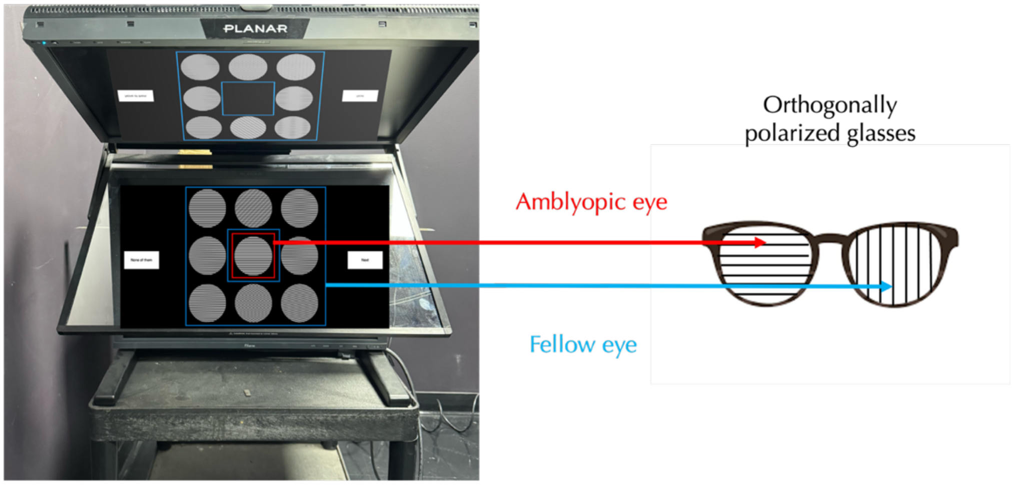

Figure E2. Dichoptic display using two monitors and a beam splitter (Planar Inc SD2620W): Images from two LCD monitors with orthogonal polarization are viewed through a beam splitter that aligns the two images for an observer. The observer wears glasses with the left eyepiece polarized to block one of the images and the right eyepiece polarized orthogonally to block the other. During the experiment, the central single grating was shown to the amblyopic eye, while the surrounding stimuli were presented to the fellow eye.

bioRxiv preprint doi: https://doi.org/10.1101/2025.06.24.661078; this version posted August 19, 2025. The copyright holder for this preprint (which was not certified by peer review) is the author/funder, who has granted bioRxiv a license to display the preprint in perpetuity. It is made available under aCC-BY 4.0 International license.

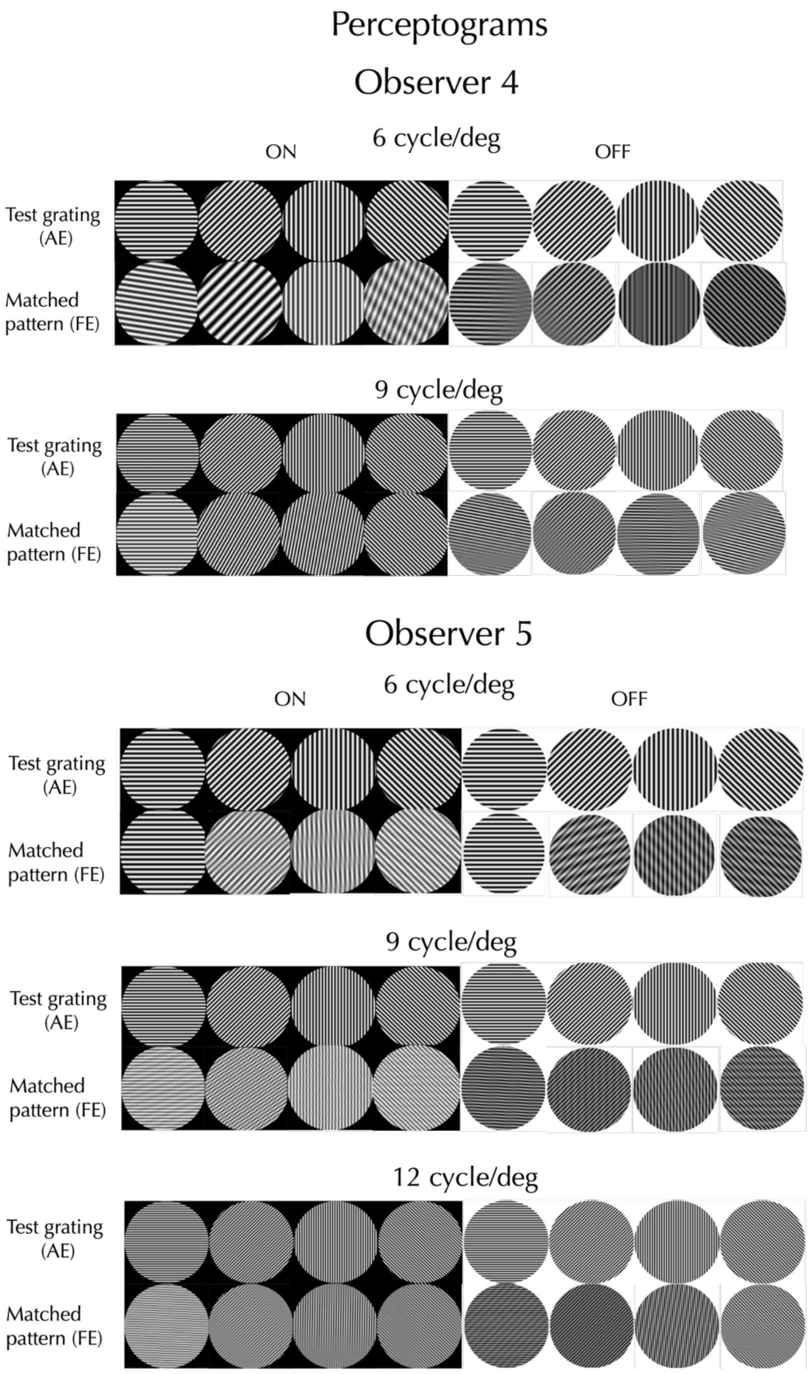

Figure E3. Perceptograms: Eight ON and OFF gratings per spatial frequency and the corresponding perceptograms are shown for Obs 4 & 5 for one session. Obs 4 could not resolve 12 cycle/deg gratings.

bioRxiv preprint doi: https://doi.org/10.1101/2025.06.24.661078; this version posted August 19, 2025. The copyright holder for this preprint (which was not certified by peer review) is the author/funder, who has granted bioRxiv a license to display the preprint in perpetuity. It is made available under aCC-BY 4.0 International license.

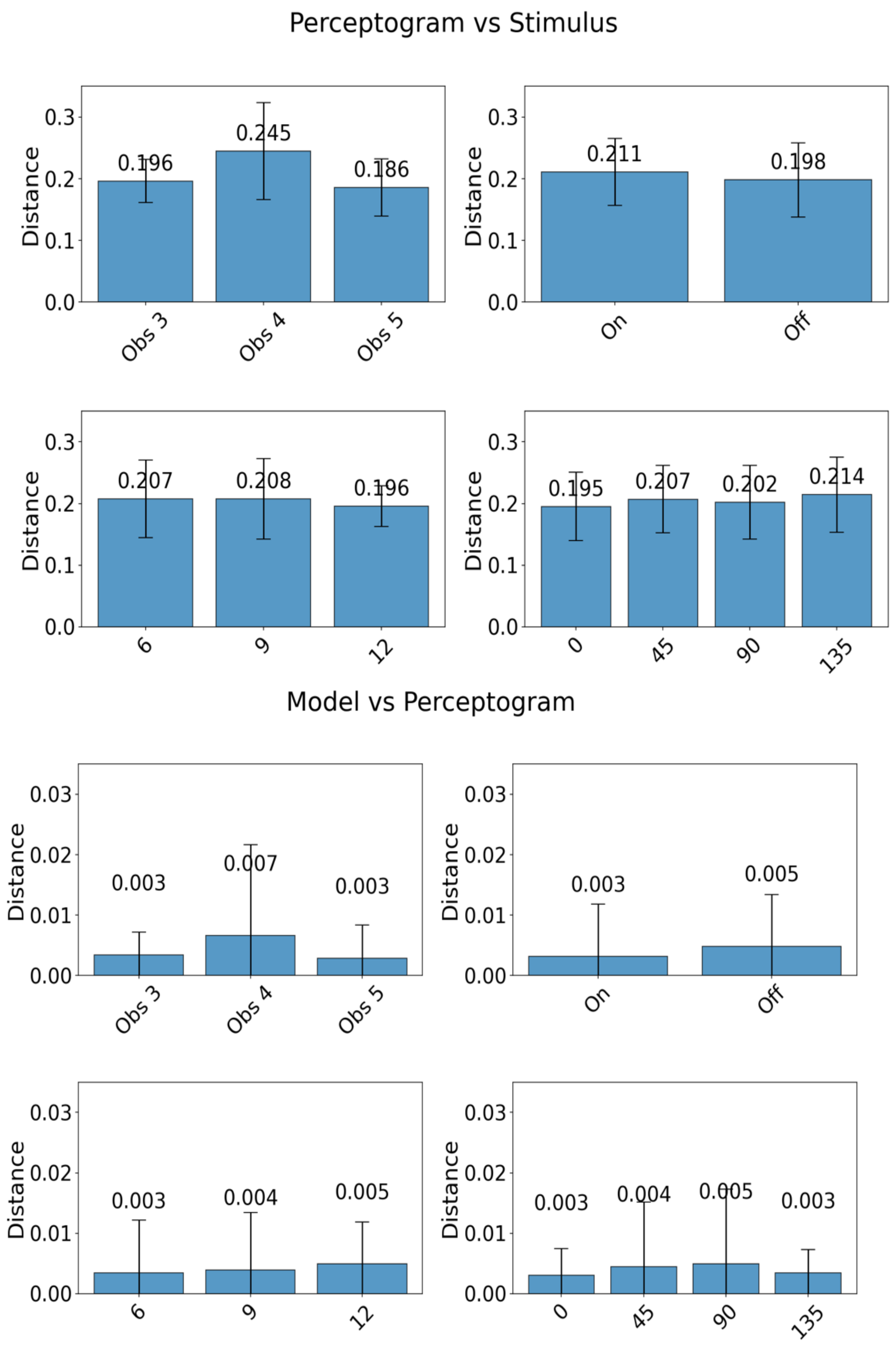

Figure E4. Comparisons of the estimated perceptual distances between images:
Rows 1 & 2: DISTS estimated perceptual distance between the measured perceptograms and stimulus gratings.
Rows 3 & 4: DISTS estimated perceptual distance between the model generated perceptograms and measured perceptograms.

bioRxiv preprint doi: https://doi.org/10.1101/2025.06.24.661078; this version posted August 19, 2025. The copyright holder for this preprint (which was not certified by peer review) is the author/funder, who has granted bioRxiv a license to display the preprint in perpetuity. It is made available under aCC-BY 4.0 International license.

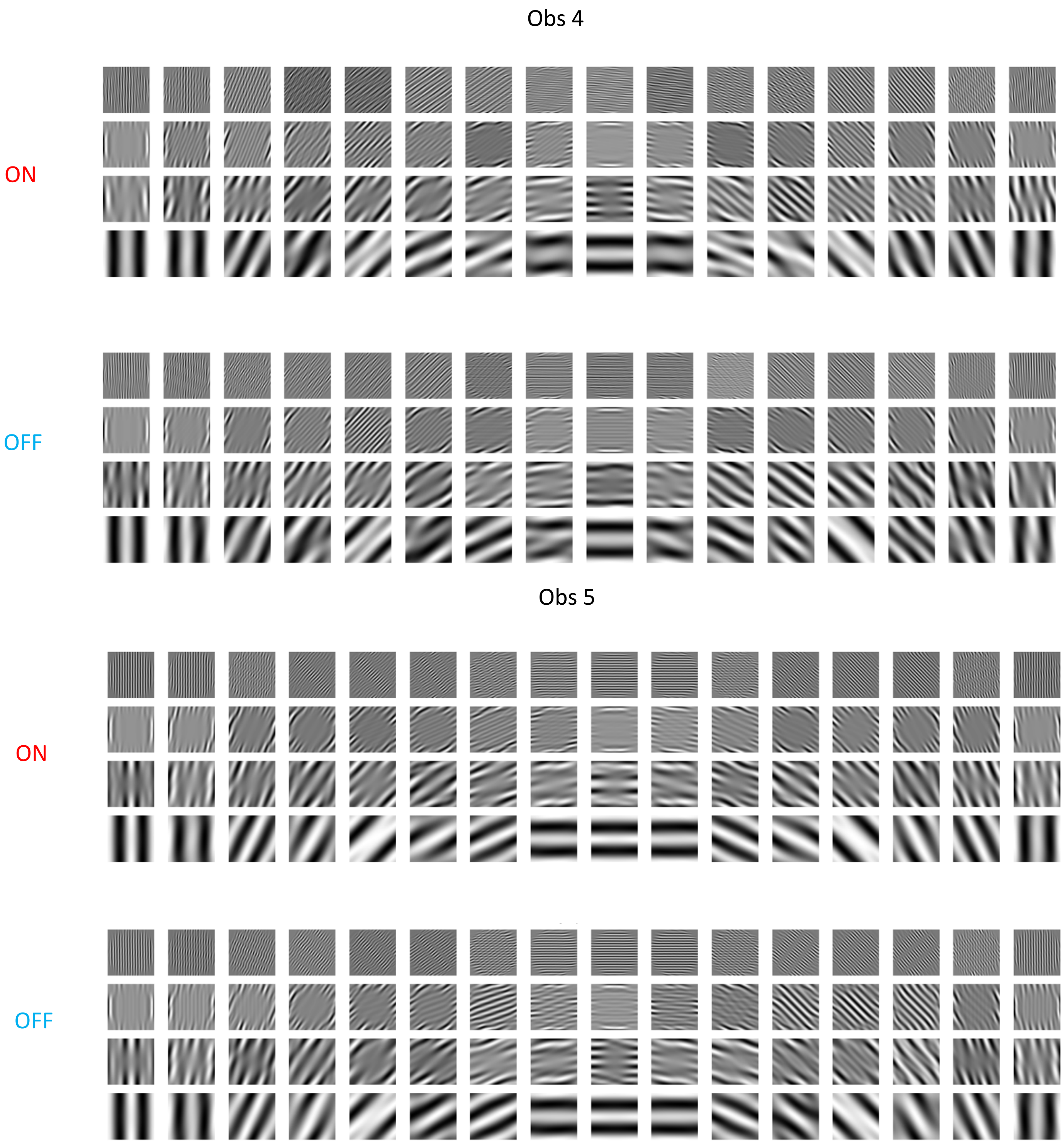

Figure E5. Transformed AE filters for ON and OFF systems:
Transformed filters for all spatial frequencies and orientations for Obs 4 and 5 who reported form distortions

bioRxiv preprint doi: https://doi.org/10.1101/2025.06.24.661078; this version posted August 19, 2025. The copyright holder for this preprint (which was not certified by peer review) is the author/funder, who has granted bioRxiv a license to display the preprint in perpetuity. It is made available under aCC-BY 4.0 International license.

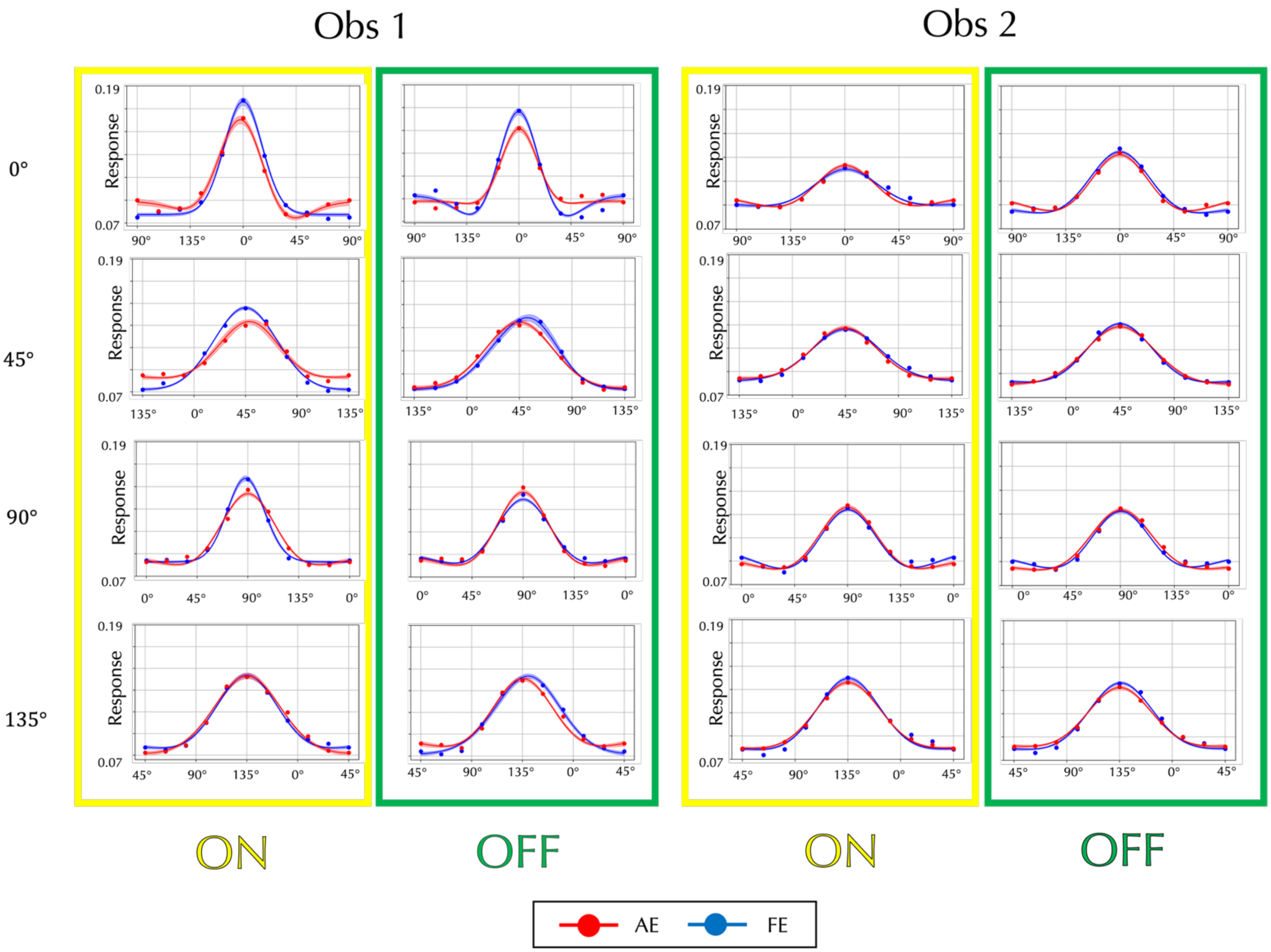

Figure E6. Perceptual Orientation-tuning curves of individuals in the Non-distortion Group:
Results for amblyopic participants who did **not** report form distortions (Dashed boxes; Yellow: ON, Green: OFF). Each row corresponds to a different target orientation. Within each panel, response magnitude is plotted against physical orientation. Response magnitude is scaled to give unit area under the curve. Red and blue dots represent the mean responses (after 5,000 bootstrap resamples) for the amblyopic and fellow eye conditions, respectively. Solid traces are the best fitting von Mises functions, with shaded areas indicating ±1 standard deviation. Most of the mean R-squared values were 0.98 or higher.

bioRxiv preprint doi: https://doi.org/10.1101/2025.06.24.661078; this version posted August 19, 2025. The copyright holder for this preprint (which was not certified by peer review) is the author/funder, who has granted bioRxiv a license to display the preprint in perpetuity. It is made available under aCC-BY 4.0 International license.

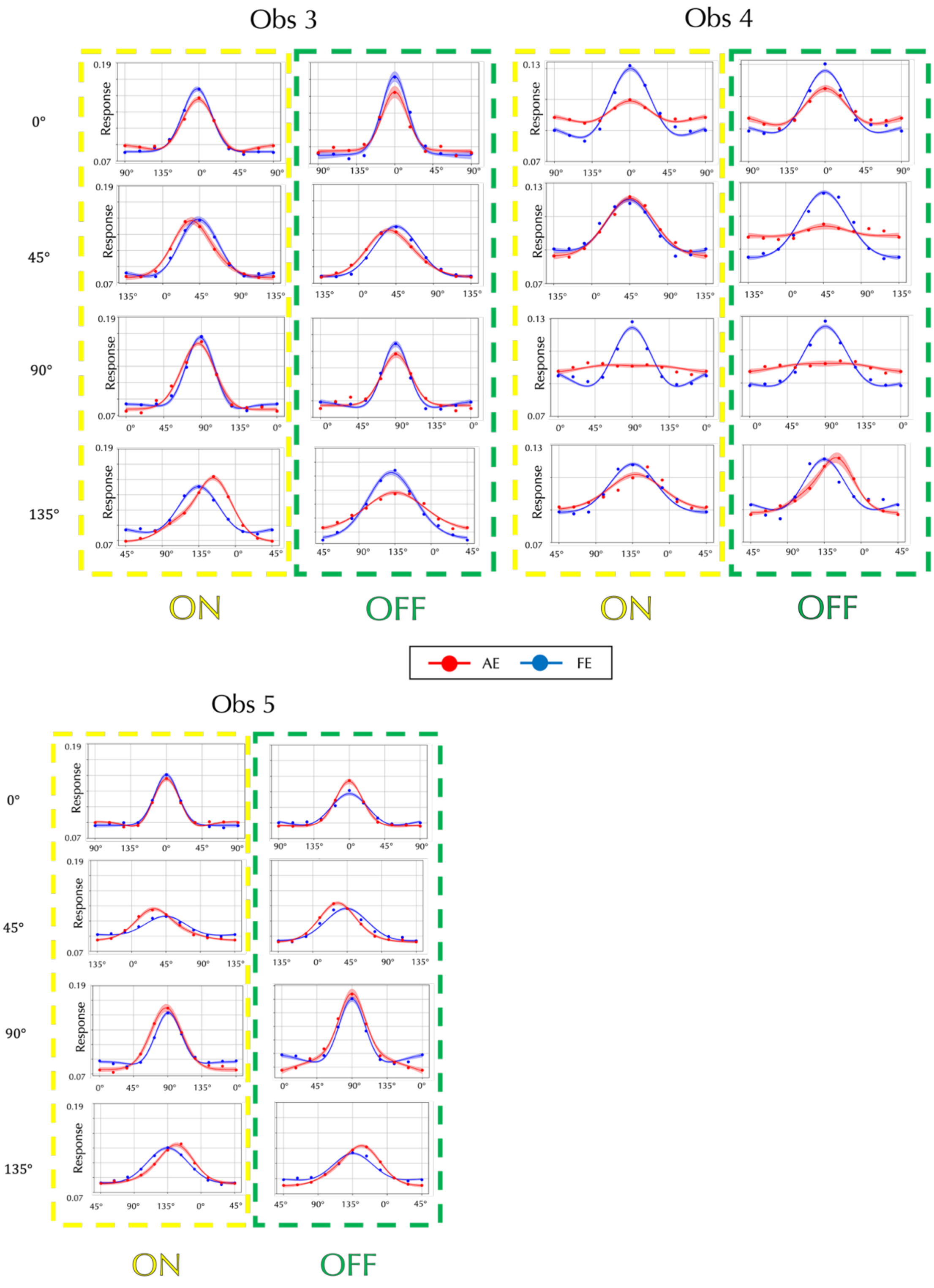

Figure E7. Perceptual Orientation-tuning curves of individuals in the Distortion Group:
Results for amblyopic participants who **did** report form distortions (Dashed boxes; Yellow: ON, Green: OFF). Each row corresponds to a different target orientation. Within each panel, response magnitude is plotted against physical orientation. Response magnitude is scaled to give unit area under the curve. Red and blue dots represent the mean responses (after 5,000 bootstrap resamples) for the amblyopic and fellow eye conditions, respectively. Solid traces are the best fitting von Mises functions, with shaded areas indicating ±1 standard deviation. Most of the mean R-squared values were 0.98 or higher.

bioRxiv preprint doi: https://doi.org/10.1101/2025.06.24.661078; this version posted August 19, 2025. The copyright holder for this preprint (which was not certified by peer review) is the author/funder, who has granted bioRxiv a license to display the preprint in perpetuity. It is made available under aCC-BY 4.0 International license.

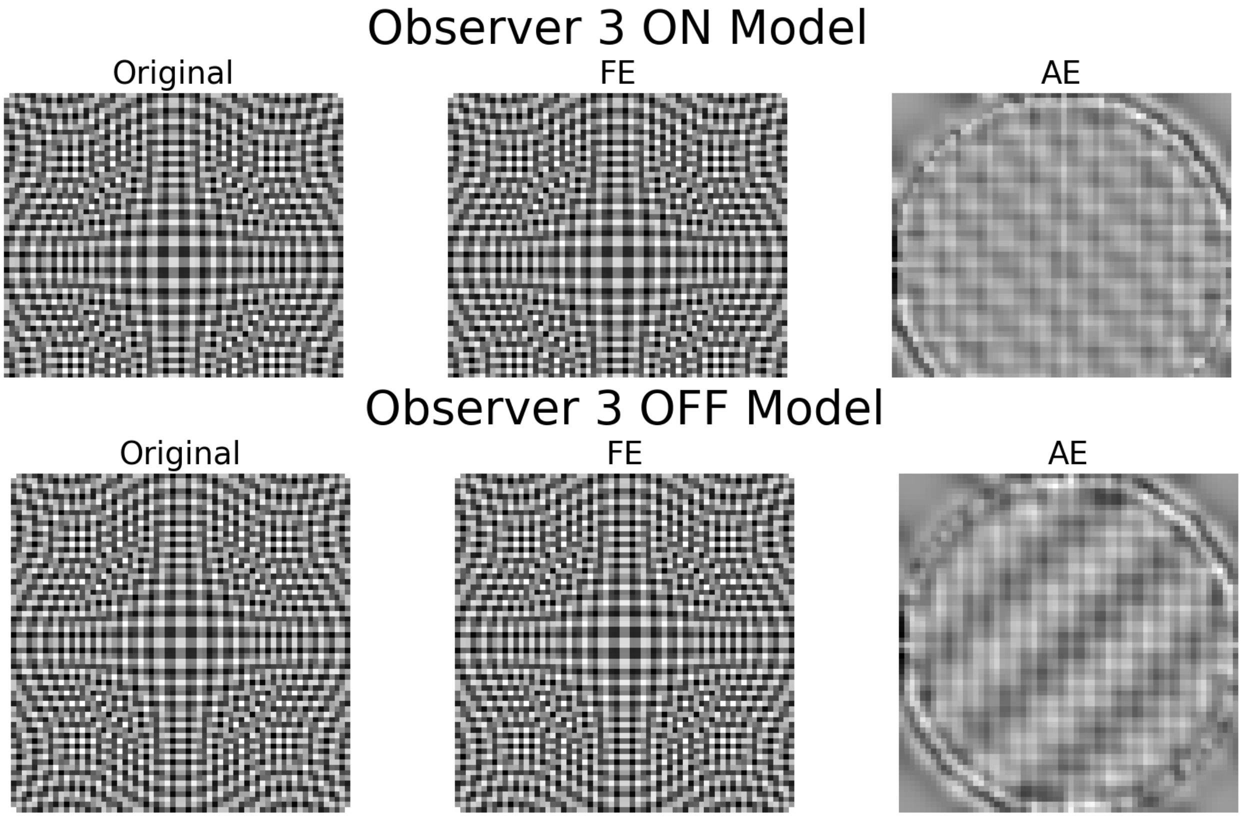

Figure E8. 3D Shape-from-Texture stimuli with reconstructions generated by the FE and AE models.
